# Cancer incidence and mortality in patients with insulin-treated diabetes: a UK cohort study

**DOI:** 10.1038/sj.bjc.6602611

**Published:** 2005-05-10

**Authors:** A J Swerdlow, S P Laing, Z Qiao, S D Slater, A C Burden, J L Botha, N R Waugh, A D Morris, W Gatling, E A Gale, C C Patterson, H Keen

**Affiliations:** 1 Section of Epidemiology, Brookes Lawley Building, Institute of Cancer Research, Sutton, Surrey SM2 5NG, UK; 2 Strathclyde Diabetic Group, Victoria Infirmary, Glasgow G42 9TY, UK; 3 Heart of Birmingham Diabetes Care, Handsworth, Birmingham B1 3AS, UK; 4 Department of Oncology, University of Leicester, Leicester LE1 6TP, UK; 5 Department of Epidemiology and Public Health, University of Leicester, Leicester LE1 6TP, UK; 6 Scottish Study Group for the Care of Diabetes in the Young, University of Aberdeen Medical School, Aberdeen AB25 2ZD, UK; 7 Royal College of Physicians of Edinburgh Group, Ninewells Hospital & Medical School, Dundee DD1 9SY, UK; 8 Department of Diabetes, Poole Hospital NHS Trust, Dorset BH15 2JB, UK; 9 Barts Oxford Study Group, Medical School Unit, Southmead Hospital, Bristol BS10 5NB, UK; 10 Department of Epidemiology & Public Health, Queen's University, Belfast BT12 6BJ, UK; 11 8 Kingsfield Road, Oxhey WD19 4T, UK

**Keywords:** insulin-treated diabetes, cohort

## Abstract

Raised risks of several cancers have been found in patients with type II diabetes, but there are few data on cancer risk in type I diabetes. We conducted a cohort study of 28 900 UK patients with insulin-treated diabetes followed for 520 517 person-years, and compared their cancer incidence and mortality with national expectations. To analyse by diabetes type, we examined risks separately in 23 834 patients diagnosed with diabetes under the age of 30 years, who will almost all have had type I diabetes, and 5066 patients diagnosed at ages 30–49 years, who probably mainly had type II. Relative risks of cancer overall were close to unity, but ovarian cancer risk was highly significantly raised in patients with diabetes diagnosed under age 30 years (standardised incidence ratio (SIR)=2.14; 95% confidence interval (CI) 1.22–3.48; standardised mortality ratio (SMR)=2.90; 95% CI 1.45–5.19), with greatest risks for those with diabetes diagnosed at ages 10–19 years. Risks of cancer at other major sites were not substantially raised for type I patients. The excesses of obesity- and alcohol-related cancers in type II diabetes may be due to confounding rather than diabetes *per se*.

Diabetes mellitus is the most common metabolic abnormality in Western populations. It occurs through two different primary disease processes, type I (insulin-dependent) and type II (non-insulin dependent), with different patient characteristics. The metabolic and hormonal antecedents and consequences of diabetes, and the treatments for it, might affect the risk of cancer. Most studies of cancer risks in patients with diabetes have related to type II diabetes. Raised risks have been found, although not entirely consistently, for endometrial, breast, renal, gallbladder and liver cancers (Kessler, 1970; Armstrong *et al*, 1976; Ragozzino *et al*, 1982; Green and Jensen, 1985; O'Mara *et al*, 1985; Adami *et al*, 1991; La Vecchia *et al*, 1994; Hjalgrim *et al*, 1997; Wideroff *et al*, 1997; Verlato *et al*, 2003), but interpretation is uncertain because of potential confounding by obesity and alcohol consumption. Pancreatic cancer risk has been found raised (Everhart and Wright, 1995), but there is uncertainty about the direction of causation, because pancreatic cancer can cause diabetes.

The aetiology of type I diabetes is not related to obesity or alcohol use, and the diabetes generally occurs well before the ages at which pancreatic cancer is prevalent. The only studies of cancer risk in patients with type I diabetes, however, have been relatively small – cohorts totalling 2400 patients (Hjalgrim *et al*, 1997) and a few case–control data (O'Mara *et al*, 1985) – and hence of limited power. Analyses of cancer risk by duration since diagnosis of diabetes could potentially illuminate causality, but cohort analyses of this factor have only been conducted for 1500 patients (Hjalgrim *et al*, 1997). We examined cancer risks in a UK cohort of 28 900 patients with insulin-treated diabetes of known date of diagnosis, most aged under 30 years at diagnosis of diabetes and therefore probably with type I diabetes.

## MATERIALS AND METHODS

The Diabetes UK (formerly British Diabetic Association (BDA)) cohort consists of 28 900 UK resident patients with insulin-treated diabetes who were under age 50 years at diagnosis of this disease. A total of 12 893 were identified in a national register of childhood cases assembled by the BDA from 1972 to 1986, and the rest were recorded in population-based geographical registers for various parts of the UK during 1972–1993, varying in their dates of data collection and the ages at diagnosis that were included. Details of the registers can be found in Laing *et al* (1999).

With appropriate ethics committee approvals, data sets from the component registers were combined, and identification details of the patients were sent to the National Health Service Central Registers (NHSCRs) for England & Wales and Scotland, and to the Central Services Agency (CSA) for Northern Ireland. The files of the NHSCRs and CSA are virtually complete population registers for their countries, and enabled the NHSCRs and CSA to provide us with ‘flagging’ information on all deaths and emigrations in the cohort up to the present. We coded cause of death using the revision of the International Classification of Diseases (ICD) in force at the time of death, and then bridge coded to ICD9 (World Health Organization, 1977). For each cohort member, we calculated person-years at risk by 5-year age group, sex, calendar year and country of residence, starting from the date of registration in the study or age 1 year, whichever was later, and ending at 30th June 2003, or the date of death, 85th birthday, emigration or other loss to follow-up, if earlier. We censored follow-up at the 85th birthday because cause of death information is relatively unreliable beyond that age. We omitted person-years and deaths under the age of 1 year because national mortality rates by subdivisions of this age group are not available, and neonatal deaths were not coded by underlying cause in England and Wales through most of the study period.

Mortality in the cohort was compared with that in the general population by calculation of standardised mortality rates (SMRs). These were calculated as the ratio of the observed number of deaths to the expected number based on application of age-, sex-, calendar year- and country-specific person-years at risk in the cohort to the corresponding mortality rates in the general population of England and Wales (for the English and Welsh cases) or Scotland (for the Scottish and Northern Irish cases). The use of Scottish data for both of the latter countries was necessary because Northern Irish data for the full period were not available to us in computer-readable form. As the two countries are fairly similar in mortality and cancer rates and Northern Ireland represented less than 5% of the subjects in the study, any consequent error will have been negligible.

Cancer incidence data for 1971 onwards are recorded on the NHSCRs (but not ‘flagged’ at the CSA), based on cancer registrations from population-based regional cancer registries. Cancer incidence data for the study cohort were therefore sent to us from the NHSCRs for the English, Welsh and Scottish cohort members. Cancer incidence was analysed similarly to mortality, by calculation of standardised incidence ratios (SIRs), with exceptions as follows: follow-up was censored at 31st December 2001 for England and Wales and 31st December 1998 for Scotland, because more recent data are not yet complete; Northern Ireland was omitted because cancer registration data for cohort members there could not be gained by flagging; and risk of nonmelanoma skin cancer was not analysed because registration of it is highly incomplete (Swerdlow *et al*, 2001).

We did not have individual data on type of diabetes, but in insulin-treated patients age at diagnosis of diabetes gives a good proxy (Laakso and Pyörälä, 1985), and it has been usual to divide analyses at age 30 years (O'Mara *et al*, 1985; Moss *et al*, 1991; Kjaer *et al*, 1992; Hjalgrim *et al*, 1997; Swerdlow *et al*, 2004) to give a younger group who are almost all type I, and an older one who are largely type II. As well as analyses for the cohort overall, therefore, we also conducted separate analyses for patients aged under 30 years at diagnosis of diabetes, of whom we calculate that at least 94% will have had type I diabetes (Laing *et al*, 1999), and for those aged 30–49 years at diagnosis, who will have been mainly type II (we estimate 64% type II and 36% type I, based on published data on insulin-dependent diabetes by age (Laakso and Pyörälä, 1985)).

The 95% confidence intervals (95% CI) and tests for trend were calculated assuming a Poisson distribution (Breslow and Day, 1987). Whether two SMRs differed was tested by an exact method (Breslow and Day, 1987). All *P*-values presented are two-sided.

## RESULTS

A total of 29 701 patients with insulin-treated diabetes diagnosed under age 50 years were identified in the component registers of the study, of whom 27 were excluded because diabetes was secondary to a congenital disease (largely cystic fibrosis), 35 because of faulty or incomplete data at registration and 334 because it was unknown whether diagnosis was at ages under 30 or 30–49 years. Of the remainder, 405 (1.4%) patients could not be traced at the NHSCRs and CSA, and the remaining 28 900 formed the study cohort. A total of 23 834 of the patients were diagnosed at ages under 30 years, and 5066 at ages 30–49 years (Table 1); 15 085 were diagnosed before 1980, 13 715 later and 100 at an unspecified date; 15 688 were male and 13 212 female subjects.

During follow-up there were 3861 deaths, 221 losses to follow-up through emigration or otherwise leaving the National Health Service, 88 patients whose follow-up was censored when they reached age 85 years, and 24 730 subjects who reached the end of follow-up alive under age 85 years; 20 676 of these latter were still under age 50 years. Follow-up was for a total of 520 517 person-years, an average of 18.0 years per subject. Of 2301 deaths in men, 228 were from cancer, and of 1560 in women, 150 were from cancer. In all, 582 cancer registrations other than nonmelanoma skin cancer were recorded in the cohort, 305 in men and 277 in women.

Table 2 shows cancer mortality in each sex in patients diagnosed with diabetes under age 30 years and in those diagnosed at 30–49 years. In the former group, there was a significant excess of deaths from cancer of the ovary (SMR 2.90; 95% CI 1.45–5.19), but not of any other cancer, including cancer of the pancreas (SMR 0.91; 95% CI 0.25–2.32). In the patients diagnosed with diabetes at ages 30–49 years, there was no raised risk of ovarian cancer (*P*=0.016 compared with the risk in the younger-onset diabetes patients), but there was a significant decrease in deaths from cancer of the nervous system (SMR 0.25; 95% CI 0.03–0.91) and nonsignificantly raised risks for several tumours previously reported at raised risk in patients with diabetes – cancers of the corpus uteri, pancreas, gall bladder and kidney (although not liver cancer). Subdividing the ovarian cancer risk more finely by age at onset of diabetes, relative risks were 2.25 (95% CI 0.06–12.52) for subjects with diabetes incident under age 10 years, 4.56 (95% CI 1.67–9.92; *P*=0.005) for diabetes at ages 10–19 years and 1.94 (95% CI 0.53–4.97) for diabetes at ages 20–29 years (not in table).

Incidence of ovarian cancer was also significantly increased in patients with diabetes of onset under age 30 years (Table 3), but there were not significantly raised or diminished SIRs for any other cancer site in this age group. Similarly to the mortality results, the SIR was greatest for those with diabetes incident at ages 10–19 years (SIR 2.90; 95% CI 1.32–5.50; *P*=0.01). The histological types of the 16 ovarian cancers occurring in this age group were six cystic, mucinous or serous, two germ cell, two adenocarcinomas, one granulosa cell, one Brenner cell and four unspecified cancers. In patients with diabetes incident at ages 30–49 years, there was significantly reduced incidence of prostate cancer and of nervous system cancer; all cases of the latter were gliomas. There was also a borderline significant excess of endometrial cancer in the same age group, but no raised risk of ovarian cancer (*P*=0.018 compared with the risk in the younger-onset diabetes patients).

Examination of cancer risks by sex (not in table) did not show any consistent patterns except that the raised pancreatic cancer risk in patients with diabetes diagnosed at ages 30–49 years was limited to men (SMR 1.72; 95% CI 0.89–3.01. SIR 1.97; 95% CI 0.98–3.53), and the lung cancer deficit in the same age group was most pronounced in women (SMR 0.58; 95% CI 0.31–0.99. SIR 0.53; 95% CI 0.28–0.90; *P*=0.02). With regard to sites for which sex differences in cancer relative risk in patients with diabetes have been reported in the literature, colorectal cancer, for which greater incidence risk in males has been reported (Will *et al*, 1998), did not show consistently greater SIRs or SMRs in males, and renal cancer, for which greater female SIRs or SMRs have been reported (Lindblad *et al*, 1999), did not show greater SIRs or SMRs in female subjects. Analyses of breast cancer risk separately at attained ages under 50 years, and 50 years and above, as a proxy for premenopausal and postmenopausal ages, were hampered by small numbers, but gave no significant results and no indication of systematically different risks by menopausal status.

When cancer mortality risks were analysed by time since onset of diabetes (Table 4), there was no trend for cancers overall. All ovarian cancer deaths in women with diabetes diagnosed under age 30 years occurred more than 10 years after diabetes diagnosis, with the greatest excess beyond 40 years (SMR 6.0; 95% CI 2.4–12.4; *P*<0.001). In the patients diagnosed with diabetes at ages 30–49 years, the modest excess of pancreatic cancer deaths did not reflect raised risk at any particular point in follow-up. Lung cancer mortality risks in patients with diabetes diagnosed at ages 30–49 years diminished significantly with longer follow-up, but this was not apparent, based on smaller numbers, for those with young-onset diabetes.

Analyses of cancer incidence risks by duration of diabetes were based on shorter follow-up (see Materials and Methods) and are therefore not shown. Lung cancer risks in patients diagnosed with diabetes at ages 30–49 years generally diminished with time, and all ovarian cancers in patients with diabetes diagnosed under age 30 years occurred more than 10 years after diabetes diagnosis.

## DISCUSSION

The most striking, and unexpected, finding of our study was the highly significant excess of ovarian cancer in women with insulin-treated diabetes diagnosed at young ages (i.e. those who presumptively had type I diabetes). There are few previous data on this risk: a nonsignificant odds ratio of 2.0 based on two cases (O'Mara *et al*, 1985) and a relative risk of 0.6 based on one case (Weiderpass *et al*, 2002b). As in previous studies (Kessler, 1970; Ragozzino *et al*, 1982; O'Mara *et al*, 1985; Adami *et al*, 1991; La Vecchia *et al*, 1994; Adler *et al*, 1996; Wideroff *et al*, 1997; Weiderpass *et al*, 2002b), we did not find a raised risk of ovarian cancer in women with older onset (i.e. largely type II) diabetes. Ovarian cancer rates might be raised in patients with diabetes via effects of hyperinsulinaemia on the ovary (Adler *et al*, 1996), but such an effect (endogenous or exogenous) could apply to type II as well as type I diabetes. Raised IGF1 levels have been proposed as a mechanism (Weiderpass *et al*, 2002b), but bioavailable IGF1 is decreased in type I diabetes (Saukkonen *et al*, 2004). Shared environmental or genetic aetiological factors for type I diabetes and ovarian cancer could in principle be an explanation, but we know of no such factors. Chance is also a possibility.

It is a limitation that we do not have individual data on menstrual or reproductive factors in our cohort, but menstrual factors in studies of women with type I diabetes have been in a direction to give diminished, not raised, risk of ovarian cancer: episodes of amenorrhoea are more frequent in women with insulin-dependent diabetes than in women in general (Kjaer *et al*, 1992); menarche is delayed in women with diabetes incident under age 10 years (Kjaer *et al*, 1992); and limited evidence suggests that menopause is considerably younger in women with childhood onset type I diabetes (Dorman *et al*, 2001). Oral contraceptive (OC) use is protective against ovarian cancer (Weiss *et al*, 1996), and there is evidence that OC use is less common in patients with diabetes (Dorman *et al*, 2001), because of potential side effects. The expected effect on ovarian cancer risk would be too small, however, to explain our findings. It is unclear whether parity differs between women with type I diabetes and other women (Dorman *et al*, 2001), but the differences would need to be very large to explain our results. We know of no evidence on oophorectomy prevalence in women with diabetes. If it were reduced this could in principle raise ovarian cancer rates, but oophorectomy rates are too low in the UK (Swerdlow *et al*, 2001) for this plausibly to explain the study findings.

There is inconclusive evidence that ovarian cancer risk may be increased by raised androgen levels (Risch, 1998), and serum androgen levels have been found raised in menstruating women with type I diabetes (Djursing *et al*, 1985). Free testosterone levels, however, are raised in women with type II diabetes (Andersson *et al*, 1994). Polycystic ovary syndrome (PCO), which includes raised androgen levels, has been associated with increased risk of ovarian cancer (Schildkraut *et al*, 1996) and with type I diabetes (Escobar-Morreale *et al*, 2000). An association of PCO also exists, however, with type II (Rajkhowa *et al*, 2000).

Type I and type II diabetes differ greatly in mean age at onset, so the restriction of ovarian cancer risk to type I might be a consequence of hormonal factors acting specifically at puberty or reproductive ages. For instance, total and free serum testosterone levels are raised in late puberty in girls with type I diabetes (Meyer *et al*, 2000), but presumably would not be abnormal at puberty in women with type II diabetes, which is incident much later. We found greatest ovarian cancer risk for women with diabetes incident around pubertal ages.

Studies of predominantly type II diabetes patients have generally shown raised risks of endometrial, renal, gall bladder, liver and pancreatic cancers, inconsistently raised risks of breast and colorectal cancers and non-Hodgkin's lymphoma, and diminished risk of prostate cancer (Kessler, 1970; Armstrong *et al*, 1976; Ragozzino *et al*, 1982; Green and Jensen, 1985; O'Mara *et al*, 1985; Adami *et al*, 1991, 1996; La Vecchia *et al*, 1994; Everhart and Wright, 1995; Zahm *et al*, 1995; Hjalgrim *et al*, 1997; Weiderpass *et al*, 1997, 2000, 2002a; Wideroff *et al*, 1997; Will *et al*, 1998; Lindblad *et al*, 1999; Verlato *et al*, 2003; Coker *et al*, 2004). Endometrial, postmenopausal breast, renal and less certainly gall bladder cancer risks have also been associated with obesity (International Agency for Research on Cancer, 2002), so the association of these cancers with type II diabetes might be a consequence of confounding by obesity. Oestrogen levels affect endometrial cancer risk (Grady and Ernster, 1996), but data are inconsistent on whether postmenopausal women with diabetes have raised oestrogen levels (Nyholm *et al*, 1989; Andersson *et al*, 1994). Possible mechanisms suggested for liver cancer in diabetes have included alcohol consumption and hepatitis (Verlato *et al*, 2003). The reduced prostate cancer risk might relate to reduced testosterone levels (Andò *et al*, 1984).

Our data for patients aged 30–49 years, who primarily had type II diabetes, showed risks raised somewhat for endometrial, liver and pancreatic cancers, slightly for renal and colorectal cancers and not raised for gall bladder or breast cancers and NHL, with reduced risk of prostate cancer. We did not find appreciably raised risks of these tumours in patients with diabetes incident aged under 30 years, that is, largely type I. Type I diabetes aetiology, unlike type II, appears unconnected with obesity or alcohol. Previous data for type I diabetes have been based on very small numbers for endometrial, renal, pancreatic, breast and prostate cancers (O'Mara *et al*, 1985; Hjalgrim *et al*, 1997; Weiderpass *et al*, 2000) and absent for gall bladder and liver cancers. Although with uncertainty from wide confidence intervals, our data are compatible with the idea that the raised risk in type II diabetes for obesity- and alcohol-related tumours may reflect confounding by obesity and alcohol, rather than aetiology by diabetes *per se*.

The diminishing risks of lung cancer mortality with longer follow-up of our older-diagnosed diabetes patients may reflect selective survival of nonsmokers, given the very high cardiovascular mortality of older patients with diabetes. The few cohort studies that have published on nervous system cancer in patients with diabetes did not find decreased risk (O'Mara *et al*, 1985; Adami *et al*, 1991; Wideroff *et al*, 1997), so the significantly reduced risk in our patients with older-diagnosed diabetes may have been a chance finding, especially as there is no obvious mechanism. Brain tumour prevalence at autopsy has been found reduced in patients with diabetes, but the deficit was solely for glioma (Aronson and Aronson, 1965), whereas in our cohort all the brain tumours were gliomas.

## Figures and Tables

**Table 1:** Cohort by age at diagnosis and year of diagnosis of diabetes, country of residence, year of entry to cohort and sex

	**Male**	**Female**	**Total**
*Age at diagnosis of diabetes (years)* a			
0–14	9503	8818	18 321
15–29	3184	2229	5413
30–49	2944	2122	5066
			
*Year of diagnosis of diabetes* a			
<1960	681	575	1256
1960–1969	1212	1027	2239
1970–1979	6272	5318	11 590
1980–1989	6150	5131	11 281
⩾1990	1316	1118	2434
			
*Country of residence*			
England and Wales	9163	7852	17 015
Scotland	6041	4860	10 901
Northern Ireland	484	500	984
			
*Year of entry to cohort*			
1972–1979	4147	3684	7831
1980–1989	7307	6077	13 384
1990–1993	4234	3451	7685
			
Total	15 688	13 212	28 900

a Omitting 100 patients diagnosed under age 30 years, for whom the exact age and year of diagnosis is not known.

**Table 2:** Cancer mortality risks by age at onset of diabetes: selected sites

		**Age at onset of diabetes (years)**			
		**<30**		**30–49**	
**ICD9 code**	**Cancer site**	**No.**	**SMR (95% CI)**	**No.**	**SMR (95% CI)**
150	Oesophagus	5	0.97 (0.31–2.26)	15	1.15 (0.64–1.90)
151	Stomach	5	0.94 (0.31–2.20)	9	0.66 (0.30–1.25)
153–154	Colon and rectum	6	0.52 (0.19–1.13)	29	1.01 (0.67–1.44)
155	Liver	1	0.50 (0.01–2.79)	4	1.01 (0.27–2.58)
156	Gall bladder	1	2.43 (0.06–13.52)	2	1.81 (0.22–6.55)
157	Pancreas	4	0.91 (0.25–2.32)	16	1.44 (0.82–2.33)
162	Lung	27	0.96 (0.63–1.39)	67	0.84 (0.65–1.06)
174, 175	Breast	10	0.74 (0.36–1.37)	17	0.86 (0.50–1.38)
179, 182	Corpus uteri	1	1.30 (0.03–7.26)	4	2.13 (0.58–5.45)
183	Ovary	11	2.90 (1.45–5.19)**	6	0.86 (0.32–1.87)
185	Prostate	1	0.30 (0.01–1.67)	9	0.73 (0.33–1.38)
188	Bladder	2	0.80 (0.10–2.90)	3	0.39 (0.08–1.14)
189	Kidney	1	0.34 (0.01–1.89)	8	1.30 (0.56–2.56)
191, 192	Nervous system	7	0.78 (0.31–1.60)	2	0.25 (0.03–0.91)*
196–199	Primary unknown	7	0.82 (0.33–1.69)	23	1.12 (0.71–1.68)
200, 202	Non-Hodgkin's lymphoma	5	0.89 (0.29–2.08)	10	1.35 (0.65–2.47)
204–208	Leukaemia	7	0.87 (0.35–1.80)	8	1.46 (0.63–2.87)
140–208	All malignancies	123	0.90 (0.75–1.08)	255	0.93 (0.82–1.05)

ICD=International Classification of Diseases; SMR=standardised mortality ratio; CI=confidence interval.

* *P*<0.05.

** *P*<0.01.

**Table 3:** Cancer incidence risks by age at onset of diabetes: selected sites

		**Age at onset of diabetes (years)**			
		**<30**		**30–49**	
**ICD9 code**	**Cancer site**	**No.**	**SIR (95% CI)**	**No.**	**SIR (95% CI)**
150	Oesophagus	4	1.06 (0.29–2.73)	15	1.49 (0.83–2.46)
151	Stomach	7	1.20 (0.48–2.47)	12	0.77 (0.40–1.35)
153–154	Colon and rectum	14	0.71 (0.39–1.19)	52	1.11 (0.83–1.46)
155	Liver	1	0.70 (0.02–3.92)	7	2.46 (0.99–5.07)
156	Gall bladder	0	—	1	0.57 (0.01–3.19)
157	Pancreas	5	1.36 (0.44–3.18)	12	1.30 (0.67–2.27)
162	Lung	27	1.14 (0.75–1.65)	56	0.81 (0.61–1.05)
172	Melanoma	20	1.21 (0.74–1.86)	9	1.20 (0.55–2.29)
174, 175	Breast	34	0.87 (0.60–1.21)	41	0.87 (0.62–1.17)
179, 182	Corpus uteri	4	1.20 (0.33–3.08)	12	1.84 (0.95–3.22)
180	Cervix	10	0.72 (0.34–1.32)	7	1.48 (0.60–3.06)
183	Ovary	16	2.14 (1.22–3.48)**	6	0.72 (0.26–1.57)
185	Prostate	6	0.84 (0.31–1.82)	15	0.57 (0.32–0.94)*
186	Testis	13	0.93 (0.49–1.59)	1	0.77 (0.02–4.27)
188	Bladder	7	0.91 (0.37–1.88)	20	1.00 (0.61–1.55)
189	Kidney	2	0.43 (0.05–1.54)	10	1.13 (0.54–2.08)
191, 192	Nervous system	14	1.02 (0.56–1.72)	2	0.26 (0.03–0.94)*
196–9	Primary unknown	5	0.65 (0.21–1.51)	15	0.81(0.45–1.34)
200, 202	Non-Hodgkin's lymphoma	14	1.13 (0.62–1.89)	12	1.00 (0.52–1.74)
204–208	Leukaemia	10	0.90 (0.43–1.65)	5	0.69 (0.22–1.61)
140–172, 174–208	All malignancies except nonmelanoma skin cancer	241	0.95 (0.84–1.08)	341	0.95 (0.85–1.05)

ICD=International Classification of Diseases; SIR=standardised incidence ratio; CI=confidence interval.

* *P*<0.05.

** *P*<0.01.

**Table 4:** Cancer mortality risks by duration since diagnosis of diabetes: selected cancer sites

		**Duration (years)**									
		**0–9**		**10–19**		**20–29**		**30–39**		**⩾40**	
**ICD9 code**	**Cancer site**	**No.**	**SMR**	**No.**	**SMR**	**No.**	**SMR**	**No.**	**SMR**	**No.**	**SMR**
*Diabetes diagnosed at ages* <*30*^a^											
162	Lung	0	—	0	—	6	1.31	8	0.90	13	0.97
174, 175	Breast	0	—	3	1.50	3	0.63	3	0.87	1	0.33
183	Ovary	0	—	1	2.16	3	3.12	0	—	7	6.01***
140–208	All malignancies^b^	9	0.92	15	0.80	30	1.02	25	0.77	43	0.95
											
*Diabetes diagnosed at ages 30–49* c											
157	Pancreas	2	3.14	3	0.99	7	1.61	2	0.77	2	3.75
162	Lung	6	1.53	21	1.00	29	0.89	11	0.58	0	—d
174, 175	Breast	1	0.40	6	0.88	6	0.93	3	0.92	1	1.53
183	Ovary	1	1.56	3	1.30	1	0.40	1	0.78	0	—
140–208	All malignancies	18	1.02	72	0.97	109	1.04	45	0.71	11	0.84

ICD=International Classification of Diseases; SMR=standardised mortality ratio.

*** *P*<0.001.

a Person-years for the duration categories shown are 176 795, 174 854, 85 680, 14 650 and 6883, respectively.

b One death in Table 2 not included here because exact duration since diagnosis is not known.

c Person-years for the duration categories shown are 15 036, 23 568, 15 218, 5497 and 817, respectively.

d Trend in risk with duration significant, *P*=0.01.
